# Validation of the Short Version (TLS-15) of the Triangular Love Scale (TLS-45) across 37 Languages

**DOI:** 10.1007/s10508-023-02702-7

**Published:** 2023-10-26

**Authors:** Marta Kowal, Piotr Sorokowski, Bojana M. Dinić, Katarzyna Pisanski, Biljana Gjoneska, David A. Frederick, Gerit Pfuhl, Taciano L. Milfont, Adam Bode, Leonardo Aguilar, Felipe E. García, S. Craig Roberts, Beatriz Abad-Villaverde, Tina Kavčič, Kirill G. Miroshnik, Izuchukwu L. G. Ndukaihe, Katarína Šafárová, Jaroslava V. Valentova, Toivo Aavik, Angélique M. Blackburn, Hakan Çetinkaya, Izzet Duyar, Farida Guemaz, Tatsunori Ishii, Pavol Kačmár, Jean C. Natividade, Ravit Nussinson, Mohd Sofian B. Omar-Fauzee, Ma. Criselda T. Pacquing, Koen Ponnet, Austin H. Wang, Gyesook Yoo, Rizwana Amin, Ekaterine Pirtskhalava, Reza Afhami, Alexios Arvanitis, Derya Atamturk Duyar, Théo Besson, Mahmoud Boussena, Seda Can, Ali R. Can, João Carneiro, Rita Castro, Dimitri Chubinidze, Ksenija Čunichina, Yahya Don, Seda Dural, Edgardo Etchezahar, Feten Fekih-Romdhane, Tomasz Frackowiak, Nasim Ghahraman Moharrampour, Talía Gómez Yepes, Simone Grassini, Marija Jovic, Kevin S. Kertechian, Farah Khan, Aleksander Kobylarek, Valerija Križanić, Samuel Lins, Tetyana Mandzyk, Efisio Manunta, Tamara Martinac Dorčić, Kavitha N. Muthu, Arooj Najmussaqib, Tobias Otterbring, Ju Hee Park, Irena Pavela Banai, Mariia Perun, Marc Eric S. Reyes, Jan P. Röer, Ayşegül Şahin, Fatima Zahra Sahli, Dušana Šakan, Sangeeta Singh, Sanja Smojver-Azic, Sinem Söylemez, Ognen Spasovski, Anna Studzinska, Ezgi Toplu-Demirtas, Arkadiusz Urbanek, Tatiana Volkodav, Anna Wlodarczyk, Mohd Faiz Mohd Y. Yaakob, Mat Rahimi Yusof, Marcos Zumárraga-Espinosa, Maja Zupančič, Robert J. Sternberg

**Affiliations:** 1 IDN Being Human Lab, University of Wrocław, Dawida 1, 50-529 Wrocław, Poland; 2 Institute of Psychology, University of Wrocław, Wrocław, Poland; 3 Department of Psychology, Faculty of Philosophy, University of Novi Sad, Novi Sad, Serbia; 4 ENES Bioacoustics Research Lab, Centre de Recherche en Neurosciences de Lyon, University of Jean Monnet Saint Étienne, Saint Étienne, France; 5 Centre National de la Recherche Scientifique, Laboratoire Dynamique du Langage, University of Lyon, Lyon, France; 6 Macedonian Academy of Sciences and Arts, Skopje, North Macedonia; 7 Crean College of Health and Behavioral Sciences, Chapman University, Orange, CA USA; 8 Department of Psychology, Norwegian University of Science and Technology, Trondheim, Norway; 9 School of Psychology, University of Waikato, Tauranga, New Zealand; 10 School of Archaeology and Anthropology, ANU College of Arts and Social Sciences, The Australian National University, Canberra, Australia; 11 School of Psychology, Central University of Venezuela, Caracas, Venezuela; 12 Division of Psychology, University of Stirling, Stirling, UK; 13 Departamento de Psiquiatría y Salud Mental, Facultad de Medicina, Universidad de Concepción, Concepción, Chile; 14 Faculty of Humanities and Education, Universidad Nacional Pedro Henríquez Ureña, Santo Domingo, Dominican Republic; 15 Department of Psychology, Faculty of Arts, University of Ljubljana, Ljubljana, Slovenia; 16 Faculty of Psychology, Saint Petersburg State University, Saint Petersburg, Russia; 17 Department of Psychology, Alex Ekwueme Federal University, Ndufu-Alike, Nigeria; 18 Institute of Psychology, Czech Academy of Sciences, Brno, Czech Republic; 19 Department of Experimental Psychology, Institute of Psychology, University of Sao Paulo, Sao Paulo, Brazil; 20 Institute of Psychology, University of Tartu, Tartu, Estonia; 21 Department of Psychology and Communication, Texas A&M International University, Laredo, TX USA; 22 Department of Psychology, Yasar University, İzmir, Turkey; 23 Department of Anthropology, İstanbul University, İstanbul, Turkey; 24 Department of Psychology and Educational Sciences, University Mohamed Lamine Debaghine Setif2, Setif, Algeria; 25 Department of Psychology, Japan Women’s University, Tokyo, Japan; 26 Department of Psychology, Faculty of Arts, Pavol Jozef Šafárik University in Košice, Košice, Slovakia; 27 Department of Psychology, Pontifical Catholic University of Rio de Janeiro, Rio de Janeiro, Brazil; 28 Department of Education and Psychology, The Open University of Israel, Raanana, Israel; 29 Institute of Information Processing and Decision Making, University of Haifa, Haifa, Israel; 30 School of Education, Universiti Utara Malaysia, Sintok, Malaysia; 31 Department of Psychology, University of Santo Tomas, Manila, Philippines; 32 Faculty of Social Sciences, Ghent University, Ghent, Belgium; 33 Department of Political Science, University of Nevada, Las Vegas, Las Vegas, NV, USA; 34 Department of Child & Family Studies, Kyung Hee University, Seoul, Republic of Korea; 35 Department of Professional Psychology, Bahria University, Islamabad, Pakistan; 36 Department of Psychology, Ivane Javakhishvili Tbilisi State University, Tbilisi, Georgia; 37 Department of Art Studies, Tarbiat Modares University, Tehran, Iran; 38 Department of Psychology, University of Crete, Rethymno, Greece; 39 Laboratoire de Psychologie Sociale, Université Paris Cité, Paris, France; 40 Department of Psychology, İzmir University of Economics, İzmir, Turkey; 41 Department of Anthropology, Hatay Mustafa Kemal University, Hatay, Turkey; 42 Center for Psychology at the University of Porto, University of Porto, Porto, Portugal; 43 Institute of Psychiatry, Psychology & Neuroscience (IoPPN), Department of Psychological Medicine, King’s College London, London, UK; 44 Institute of Psychology, Vilnius University, Vilnius, Lithuania; 45 Department of Psychology, University of Buenos Aires, Buenos Aires, Argentina; 46 Department of Psychology, Centro Interdisciplinario de Psicología Matemática y Experimental, Buenos Aires, Argentina; 47 Department of Education, International University of Valencia, Valencia, Spain; 48 Department of Psychiatry Ibn Omrane, Razi Hospital, Manouba, Tunisia; 49 Faculty of Medicine of Tunis, Tunis El Manar University, Tunis, Tunisia; 50 School of Psychological Sciences, University of Melbourne, Melbourne, Australia; 51 Department of Psychosocial Science, University of Bergen, Bergen, Norway; 52 Cognitive and Behavioral Neuroscience Laboratory, University of Stavanger, Stavanger, Norway; 53 Department of Marketing Management and Public Relations, Faculty of Organizational Sciences, University of Belgrade, Belgrade, Serbia; 54 Department of Organization, Management, and Human Resources, ESSCA School of Management, Paris, France; 55 Institute of Education & Research, Women University Mardan, Mardan, Pakistan; 56 Department of Pedagogy, University of Wrocław, Wrocław, Poland; 57 Department of Psychology, Faculty of Humanities and Social Sciences, J.J. Strossmayer University of Osijek, Osijek, Croatia; 58 Department of Psychology, Ivan Franko National University of Lviv, Lviv, Ukraine; 59 Cognition, Langues, Langage, and Ergonomie, University of Toulouse, Toulouse, France; 60 Department of Psychology, Faculty of Humanities and Social Sciences, University of Rijeka, Rijeka, Croatia; 61 Department of Psychology and Counselling, Universiti Tunku Abdul Rahman, Kampar, Malaysia; 62 Department of Applied Psychology, National University of Modern Languages, Islamabad, Pakistan; 63 Department of Strategy, University of Agder, Kristiansand, Norway; 64 Department of Child and Family Studies, Yonsei University, Seoul, Korea; 65 Department of Psychology and Psychotherapy, Witten/Herdecke University, Witten, Germany; 66 Institute of Sport Professions, University of Ibn Tofail, Kenitra, Morocco; 67 Department of Psychology, Faculty of Law and Business Studies Dr Lazar Vrkatić, Union University, Novi Sad, Serbia; 68 Department of Strategy and Management, University of Agder, Kristiansand, Norway; 69 Department of Psychology, Manisa Celal Bayar University, Manisa, Turkey; 70 Department of Psychology, University of Ss. Cyril and Methodius, Trnava, Slovakia; 71 Department of Psychology, Ss. Cyril and Methodius University in Skopje, Skopje, Republic of North Macedonia; 72 Department of Humanities, Icam School of Engineering, Toulouse Campus, Toulouse Cedex, France; 73 Psychological Counseling and Guidance, Mef University, İstanbul, Turkey; 74 Department of Pedagogy and Psychology, Kuban State University, Krasnodar, Russia; 75 Escuela de Psicología, Universidad Católica del Norte, Antofagasta, Chile; 76 Psychology Career, Salesian Polytechnic University, Quito, Ecuador; 77 Department of Psychology, Cornell University, Ithaca, NY USA

**Keywords:** Triangular theory of love, Triangular Love Scale, Cross-cultural

## Abstract

Love is a phenomenon that occurs across the world and affects many aspects of human life, including the choice of, and process of bonding with, a romantic partner. Thus, developing a reliable and valid measure of love experiences is crucial. One of the most popular tools to quantify love is Sternberg’s 45-item Triangular Love Scale (TLS-45), which measures three love components: intimacy, passion, and commitment. However, our literature review reveals that most studies (64%) use a broad variety of shortened versions of the TLS-45. Here, aiming to achieve scientific consensus and improve the reliability, comparability, and generalizability of results across studies, we developed a short version of the scale—the TLS-15—comprised of 15 items with 5-point, rather than 9-point, response scales. In Study 1 (*N* = 7,332), we re-analyzed secondary data from a large-scale multinational study that validated the original TLS-45 to establish whether the scale could be truncated. In Study 2 (*N* = 307), we provided evidence for the three-factor structure of the TLS-15 and its reliability. Study 3 (*N* = 413) confirmed convergent validity and test–retest stability of the TLS-15. Study 4 (*N* = 60,311) presented a large-scale validation across 37 linguistic versions of the TLS-15 on a cross-cultural sample spanning every continent of the globe. The overall results provide support for the reliability, validity, and cross-cultural invariance of the TLS-15, which can be used as a measure of love components—either separately or jointly as a three-factor measure.

## Introduction

What is love? Millions of people have asked this question, as exemplified by popular songs, best-selling books, and informal exchanges of opinions and love experiences with close friends (de Rougemont, 1983; Werner, 2012). Nevertheless, despite it being one of the most commonly used words throughout human culture and history (Bode & Kushnick, 2021), there is no simple definition of love. Indeed, the topic of love has long evoked heated discussions among scholars attempting to coin a standard and widely accepted definition (Bode & Kushnick, 2021). The matter becomes even more complex when we consider that there may be many types of love beyond romantic love, such as parental, sisterly, brotherly, platonic, or friendly love (Fehr, 1994). Herein, we narrow the scope of our investigation to romantic love—that is, overly simplifying, love felt for a romantic partner or mate, whether actual or desired.

Sternberg’s (1988, 2021) Triangular Theory of Love is currently one of the most prominent theories of love (Clemente et al., 2020). It contends that love consists of three components: intimacy, passion, and commitment. Intimacy is associated with feelings of warmth, communication, and connectedness. High intimacy toward one’s partner implies that the relationship is close, caring, reflective of good communication, and demonstrates feelings of connectedness. Passion refers to feelings of excitement, desire, attraction, and physical arousal felt in the presence of a loved one. Commitment, the most cognitive component of love, pertains to one’s conscious decision and motivation to maintain the relationship. Commitment is often treated as a relatively “cold” component of love. High commitment refers to one’s belief that the given relationship can last long into the future (Sternberg, 1988).

It is worth noting that there are several other overarching theories of romantic love. One classic typology proposes six distinct love styles: Agape, Eros, Ludus, Mania, Pragma, and Storge (Hendrick & Hendrick, 1986; Lee, 1977). Another distinguishes between two types of romantic love: passionate and companionate (Feybesse & Hatfield, 2019; Hatfield & Walster, 1978). This is complemented by two recent theories. Fletcher et al. (2015) drew on biological and behavioral explanations of romantic love, pointing to the evolutionary rationale for love’s existence and highlighting its universality across cultures. Bode and Kushnick (2021) aimed to inform various scientific fields (e.g., biology, humanities) and proposed a more holistic notion of romantic love, disentangling its functions, expressions, and origins.

With so many theories of love (for a review, see Karandashev, 2022), numerous scales to measure love have been created. One of the first love scales was developed by Rubin (1970), who differentiated liking from loving, with the latter consisting of caring, dependence, and mutual exclusiveness (for further discussion of the differentiation between liking and loving, see Sternberg, 1987). Hatfield and Sprecher (1986) developed a scale measuring passionate love (the passionate love scale, PLS). Another scale (the love attitudes scale, LAS) measures the six distinct love styles described above (i.e., Agape, Eros, Ludus, Mania, Pragma, Storge; Hendrick & Hendrick, 1986). Sprecher and Metts (1989) created the Romantic Beliefs Scale (RBS), which tests the romantic ideology of love, consisting of four beliefs about love: (1) love finds a way, (2) one and only, (3) idealization, and (4) love at first sight. For a fuller review of more than 30 different love scales, see Hatfield et al. (2012).

Here, we focused on the Triangular Love Scale (TLS-45; Sternberg, 1997), one of the most widely used love scales (Hatfield et al., 2012). The original TLS-45 captures the three components of love proposed in the triangular theory of love (Sternberg, 1988)—that is, intimacy, passion, and commitment—each measured by 15 items. The TLS-45 stands out from other love scales because it was recently validated in a large-scale study across 25 countries (and 19 languages) by Sorokowski et al. (2021). It showed adequate psychometric properties across different cultural contexts. In particular, the authors provided evidence for a good overall fit of the scale’s three-factor structure and very high composite reliabilities of the latent factors. Furthermore, the TLS-45 was invariant across countries (including configural, metric, and scalar invariance).

Despite these positive attributes, there is one crucial pitfall of using the TLS-45: it has been employed in a non-standardized and non-systematic manner. Indeed, many scholars have truncated the original scale without consistent standardization, consensus, or validation of the shortened versions. To illustrate, our extensive review of the literature spanning from 1997 (the publication date of the original validation of the TLS-45; Sternberg, 1997) to 2021 identified 232 studies that used some version of the scale. Only a minority of the studies (*N* = 83, 36%) used the complete and original scale, whereas most (*N* = 145, 64%) used varied and—most frequently—shortened versions. This finding suggests that most scholars consider the original scale too long and time-consuming for participants.

The great variety of shortened adaptations bears many risks and challenges for researchers. First, shortened scales are not always validated, potentially yielding unreliable and less than fully valid data (Morgado et al., 2018). Second, the implemented changes (e.g., in the number of items or the response options) limit the ability of researchers to make valid comparisons across studies. This, in turn, limits the generalizability of the results. Third, substantial changes in the framing of items might jeopardize a theory-driven construct of latent love, raising concerns about whether the original and the altered scales assess the same dimension. If there are differences across studies, they may be due to the construct, to the measurement, or to both.

The frequent use of adapted triangular love scales indicates high demand for a brief, reliable, and valid three-component love scale that could be used widely and cross-culturally. Weighting the importance of validating measures in science to promote its advancement (Canan et al., 2020; Kiekens et al., 2022; Plaza-Vidal et al., 2021), we aimed to develop a short version of the triangular love scale with robust psychometric properties. For this goal, we performed four consecutive studies to compile and assess a short version of the TLS.

In Study 1 (*N* = 7,332), we re-analyzed secondary data from a large-scale multinational study that validated the original TLS-45 (Sorokowski et al., 2021) to establish whether the scale could be truncated. In Study 2 (*N* = 307), we conducted a pilot analysis with a 15-item version of the TLS (TLS-15) to test if its psychometric properties were similar to those of the original scale. In Study 3 (*N* = 413), we implemented a repeated-measures design to assess the test–retest reliability of the TLS-15; we also tested the convergent validity of the TLS-15. In Study 4, we used the TLS-15 in a large-scale global investigation conducted on 60,311 participants from 156 countries to establish whether the resulting scale was invariant across 37 linguistic versions and had robust psychometric properties.

## Study 1

### Method

In Study 1, we re-analyzed data from a previous large-scale collaboration (Sorokowski et al., 2021), in which the authors validated the TLS-45 using data from 25 countries.

### Participants

We followed Sorokowski et al.’s (2021) inclusion criteria (i.e., non-single individuals from countries with sample sizes of at least 150). Thus, our sample consisted of 7332 individuals from 25 countries (i.e., Algeria, Australia, Belgium, Brazil, Cuba, Estonia, Croatia, Hungary, India, Italy, Lithuania, the Netherlands, Pakistan, Poland, Portugal, Romania, Russia, Serbia, Slovakia, Slovenia, Spain, Turkey, Uganda, Uruguay, and Vietnam), among whom 4,028 self-identified as female (55%), 3,288 as male (45%), and 16 individuals (0.002%) who did not report their sex. Ages ranged from 18 to 76 years (*M* = 30.67, *SD* = 11.10, *Mdn* = 27). There were 3620 dating individuals (49%), 2821 were married (39%), and 891 were engaged to be married (12%). Out of the available information (3466, 47% of the total sample), 1973 participants (57%) were recruited from a community sample, and 1493 individuals from a university student sample (43%). Furthermore, 15 participants (0.2%) indicated having no formal education, 106 (1%) completed only up to primary school, 1094 (15%) completed only up to secondary school, 2162 individuals (30%) completed only up to high school, 3822 (52%) attained a post-secondary degree, and 133 participants (2%) did not respond to the question about their education level.

### Procedure

Data were collected at all study sites roughly simultaneously and in-person, using either a paper–pencil method or by completing the questionnaire on a computer with the assistance of a researcher. Participants first gave informed consent. They then completed a set of questionnaires, including the TLS-45 (Sorokowski et al., 2021, 2023), marital satisfaction (Kowal et al., 2021; Sorokowski et al., 2019), mate preferences (Walter et al., 2020), and social media (Kowal et al., 2020b). For more details on sampling and procedure, see Sorokowski et al. (2021).

### Measure

Participants filled out the original Triangular Love Scale (TLS-45; Sternberg, 1988, 1997), consisting of 45 items (i.e., 15 items comprising each of the Intimacy, Passion, and Commitment subscales). An exemplary item from each subscale reads: “I share deeply personal information about myself with my partner” (Intimacy); “I fantasize about my partner” (Passion); and “I view my relationship with my partner as permanent” (Commitment). Responses ranged from 1–*not at all* to 9–*extremely*.

### Statistical Analyses

In the first step, we computed average scores of the three love subscales (i.e., intimacy, passion, and commitment). Then, we assessed the normality of the subscales by examining kurtosis and skewness values. We followed recommended guidelines for large samples, including univariate kurtosis values not larger than |7| and skewness values not larger than |2| (Kim, 2013). In the following step, we assessed inter-item correlations as a measure of dimensionality, item-subscale correlations as a measure of discrimination properties, and internal reliabilities (i.e., alpha, omega total, and omega hierarchical; Hayes & Coutts, 2020).

We then performed confirmatory factor analyses (CFA) with weighted least squares with adjusted means (WLSM) estimators to test the TLS structure. We tested each love subscale separately and jointly in the three-factor model. We assessed model fit by inspecting a Comparative Fit Index (CFI) and Tucker-Lewis Index (TLI), which should be above 0.95, a root-mean-square error of approximation (RMSEA), which should be below 0.08, and a Standardized Root-Mean-Square Residual (SRMR), which should be below 0.06 to indicate a good model fit (Hu & Bentler, 1999). We then checked for item loadings within each subscale to identify the items with the highest loadings. We did not test for the equivalence of invariance across countries, as this was already established by Sorokowski et al. (2021).

Subsequently, we tested the psychometric properties of the TLS-45 using item response theory (IRT) analysis. First, we checked for the IRT assumptions: unidimensionality, local independence, and monotonicity. We evaluated the dimensionality of the TLS-45 and its subscales with three methods: Velicer’s (1976) minimum average partial (MAP) criterion, parallel analysis (Hayton et al., 2004), and the ratio of the first to second eigenvalues (with a recommended threshold greater than 4:1; Slocum-Gori & Zumbo, 2011). Local independence was investigated with adjusted Q3 statistics (aQ3; Marais, 2013), which depict residual correlations after accounting for the influence of the common latent factors. When evaluating dependency, we followed standard guidelines, in which adjusted Q3 values above |0.20| are flagged as violations of local independence (Christensen et al., 2017). Monotonicity was investigated via visual inspection of the item characteristic curve (ICC) and insight into the order of thresholds. If thresholds were disordered, the response scale should be shortened (Silvia & Rodriguez, 2020). Then, we computed a two-parameter generalized partial credit model (GPCM) using marginal maximum likelihood.

We investigated items’ mean-square infit (inlier-sensitive or information-weighted fit) and outfit (outlier-sensitive fit) statistics, which present a good fit if close to 1.00 (Bond et al., 2020). We also evaluated the root mean squared deviation (RMSD) statistics. RMSD statistics above 0.08 indicate high misfit, those between 0.05 and 0.08 indicate medium misfit, those below 0.05 indicate small misfit, and those below 0.02 show negligible misfit (Köhler et al., 2020). In the last step, we estimated the expected a posteriori score (EAP) as a measure of reliability, difficulty, and discrimination parameters for all items, and the total information function for all subscales and total TLS-45 score, with the minimal acceptable value of 0.70 indicating good reliability (Taber, 2018). Difficulty (*β*) refers to the amount of the latent trait necessary to have a 50% chance of endorsing the item, discrimination (*a*) refers to the capability of an item to determine people at different levels of latent trait (Baker & Kim, 2017), and information refers to the reliability or precision of measurement at each level of the latent trait. All analyses were performed in R (4.1.0), and all packages used in Study 1 are listed in the Supplementary Material (SM).

### Results

Table S1 in the SM presents means, standard deviations, skewness and kurtosis values, and reliability scores of the three love subscales and the total TLS-45 score. Table S2 (SM) shows all item means, standard deviations, skewness, and kurtosis values. In summary, most of the TLS-45 items (except for Items 4, 10, 31, 32, and 42) had skewness and kurtosis values within the expected range of normal distribution. Furthermore, we found evidence for the good reliability of the three love subscales (Table S1 in SM). Items-total correlations for subscales were significant and varied from 0.62 to 0.81 in the case of Intimacy, 0.67 to 0.79 for Passion, and 0.70 to 0.86 for Commitment, which indicates good item discrimination. Table S3 (SM) shows all item-total correlations for the subscales and the total TLS-45 score, while Table S4 (SM) shows inter-item correlations that varied from *r* = 0.20–0.77, potentially indicating some redundancy (Streiner et al., 2015).

Confirmatory factor analyses yielded similar results for all three subscales separately and jointly in the three-factor model. CFI and TLI values were above 0.95, while RMSEA was below 0.08, and SRMR was below 0.06, which indicates a good model fit (see Table S5 in the SM for detailed results of the CFA). Items’ loadings were high and fell between 0.57 and 0.84 (*M* = 0.74, *SD* = 0.06, *Mdn* = 0.74, see Table S6 in the SM). Items that had the highest loadings were 10, 11, 6, 9, 14, 2 (Intimacy subscale), 25, 28, 21, 26, 18, 17, 19 (Passion subscale), and 38, 41, 44, 40, 42, 34 (Commitment subscale). The correlations between the three factors were substantial: *r* = 0.75 between intimacy and passion, *r* = 0.81 between intimacy and commitment, and *r* = 0.81 between passion and commitment.

Next, we proceeded with the IRT analyses. First, we analyzed unidimensionality. Although the parallel analyses suggested six factors for intimacy, five factors for passion, and five factors for commitment, the first factor was clearly dominant for each subscale, with a noticeably higher eigenvalue compared with the rest of the factors. The Velicer’s MAP criterion indicated one factor within Intimacy, two factors within passion, one factor within commitment, and four factors within the TLS. However, the ratio of the first to second eigenvalues greater than 4:1 provided evidence for a one-factor structure of all three subscales analyzed separately. Adjusted Q3 statistics (aQ3) indicated that seven items of intimacy (five pairs), eight items of Passion (seven pairs), and ten items of commitment (five pairs) might be considered locally dependent. We then investigated monotonicity, that is, whether a 9-point scale is ordered. The evaluation of the thresholds revealed that all items were disordered, which strongly suggested that the response scale should be shortened (Silvia & Rodriguez, 2020). Figures S1–S45 in the SM show item-characteristic curves.

Item parameters and infit and outfit characteristics are presented in Tables S7–S8, respectively. Results showed that items had similar and above 1 mean-square infit statistics, which indicated no presence of misfit among average-difficulty items (Bond et al., 2020). However, the items differed regarding the outfit statistics. In the case of Intimacy, items’ outfit statistics ranged from 0.91 to 1.12; for Passion, they ranged from 0.98 to 1.16; and for Commitment, they ranged from 0.76 to 1.28, indicating that there might be more outliers and unusual responses in the Commitment subscale (Wu & Adams, 2013). The evaluation of the root mean squared deviation (RMSD) statistics provided evidence for the proper fit of the items. All items’ RMSD statistics were below 0.05 for Intimacy and ranged between 0.01 and 0.03. RMSD statistics ranged between 0.02 to 0.03 for Passion and 0.02 to 0.04 for Commitment. Items with the lowest RMSD were: 14, 10, 11, 15, 2 (Intimacy subscale); 19, 18, 28, 17, 16 (Passion subscale); 38, 34, 44, 41, 42 (Commitment subscale; see Table S9 in the SM for detailed statistics of each item).

Accordingly, the analyses of items’ difficulty and discrimination are presented in Figures S46 and S47 in the SM. The endorsement of all items is high. Most of the *β* parameters fell below 0 theta (ranging from –2.01 to 0.65), meaning that the probability for item endorsement and choosing responses 8 or 9 (*extremely*) as an answer required only an average level of the latent trait and not an above-average level. Passion was the most difficult subscale; the endorsement of items was the lowest. It is noteworthy that all items were mostly easy, while Intimacy and Commitment were less difficult—that is, endorsement of their items was higher. The item discrimination analysis revealed that most discrimination parameters were reasonably high, ranging from 0.50 to 2.08 (Baker & Kim, 2017). The items that had the highest difficulty from the Intimacy subscale were: 13, 12, 6, 11, 8; the Passion subscale: 20, 30, 29, 23, 18; the Commitment subscale: 39, 35, 36, 41, 34. The items that had the highest discrimination from the Intimacy subscale were: 10, 9, 11, 6, 14; the Passion subscale: 28, 25, 19, 17, 21; the Commitment subscale: 44, 38, 41, 40, 42.

Test-information functions revealed that the subscales differed regarding how much information they provided and at which trait levels they were the most reliable (see Figure S48 in the SM). The Commitment and Intimacy subscales provided much more information than the Passion subscale, but they did so at a lower trait level than for Passion. In brief, all items seemed to carry the most information at the below-average level of the latent trait continuum. At the same time, all items made a substantially weaker distinction between the average and above-average levels of the latent trait. Total information function graphs show that the reliability of each of the subscales was above the minimal acceptable value of 0.70 (Taber, 2018), between –3 and 1.5 standard scores in the case of Intimacy and Commitment, and between –3 and 2 in the case of Passion (see Figures S49-S51 in the SM).

### Discussion

The results of the CFA analyses provided evidence for the three-factor structure of the TLS-45 and satisfactory reliability coefficients. However, the scale’s monotonicity analysis revealed that the items’ thresholds were disordered, signifying that the probability of selecting certain response options deviates from the expected order. In other words, participants might face difficulties when discriminating between so many response categories, which negatively impacts the accuracy of the latent trait’s measurement. Thus, we decided to substantially truncate the response scale and retain only those response categories that exhibited a consistent upward trend in the endorsement proportion along with the increase of the underlying construct. We balanced capturing nuanced distinctions of the latent trait levels with minimizing the participants’ cognitive burden when choosing an appropriate response category by shortening the response scale from 9-points to 5-points (from 1–*not at all* to 5–*extremely*).

Moreover, when we focused on specific item parameters, some item characteristics were less satisfactory than others. Table 1 shows which items had the worst psychometric properties, such as the highest kurtosis values and item-total correlations across two or three subscales simultaneously, the lowest loadings, the highest infit and outfit, and RMSD statistics, the lowest discrimination and difficulty parameters. Weighing the advantages and disadvantages of items based on the above criteria, we chose five items from each of the scales that had the best psychometric properties to propose a short version of the TLS-45—that is, the TLS-15. In the Intimacy subscale, these items were 2, 6, 9, 11, 13, in the Passion subscale, these items were 18, 19, 21, 25, 28, and in the Commitment subscale, these items were 34, 38, 40, 41, 43 from the original TLS-45. In the subsequent study, we tested the psychometric properties of the TLS-15 in an independent sample.

**Table 1:** Psychometric properties of the TLS-45 and selection of items for the TLS-15

Subscale	Item	Kurtosis and item-total correlations	CFA loadings	Infit and Outfit misfit	RMSD misfit	Discrimination	Difficulty
Intimacy							
	1		*x*		*x*	*x*	*x*
	**2**						
	3	*x*					*x*
	4	*x*	*x*	*x*		*x*	*x*
	5		*x*			*x*	*x*
	**6**						
	7						*x*
	8					*x*	
	**9**			*x*			*x*
	10	*x*					*x*
	**11**						
	12		*x*			*x*	
	**13**					*x*	
	14	*x*					*x*
	15		*x*			*x*	
Passion							
	16		*x*			*x*	*x*
	17					*x*	*x*
	**18**					*x*	
	**19**						*x*
	20		*x*	*x*	*x*	*x*	
	**21**					*x*	
	22	*x*				*x*	*x*
	23	*x*	*x*			*x*	
	24	*x*	*x*	*x*	*x*	*x*	*x*
	**25**						
	26					*x*	*x*
	27	*x*	*x*		*x*	*x*	*x*
	**28**					*x*	*x*
	29		*x*	*x*	*x*		
	30		*x*	*x*	*x*	*x*	
Commitment							
	31	*x*	*x*		*x*	*x*	*x*
	32	*x*		*x*			*x*
	33	*x*	*x*	*x*	*x*	*x*	*x*
	**34**						
	35				*x*		
	36				*x*	*x*	
	37				*x*		
	**38**						
	39			*x*	*x*	*x*	
	**40**			*x*	*x*		*x*
	**41**			*x*			
	42	*x*		*x*			*x*
	**43**			*x*	*x*		*x*
	44	*x*		*x*			*x*
	45	*x*		*x*		*x*	*x*

Italicized ‘x’s depict the items with the poorest performance in each of the categories. Items present in bold are those that were chosen for the TLS-15

## Study 2

### Method

Study 2 aimed to investigate whether the short version of the Triangular Love Scale (TLS-15) preserves similar psychometric properties to those of the original scale (TLS-45).

### Power Analysis

Power analysis was conducted to determine the number of participants needed for Study 2. Using R (version 4.0.1) and the packages *lavaan* (Rosseel, 2012), *semPlot* (Epskamp, 2015), *semTools* (Jorgensen et al., 2018), and *tidyr* (Wickham & Henry, 2019), a power analysis for structural equation modeling was performed. Based on a Monte Carlo analysis (Metropolis & Ulam, 1949; Muthén & Muthén, 2002) with 5000 iterations, we detected that a sample size of 300 individuals is needed to obtain a power of 0.95 for a CFA, with an alpha of 0.05 and fixed parameter settings for the first loadings of each of the three love subscales and their high covariance (at a minimum of 0.8). This is consistent with the rule of thumb of about 20 participants per item (Kyriazos, 2018; Wang & Rhemtulla, 2021; Wolf et al., 2013).

### Participants

We recruited 307 Polish participants, 150 men (49%) and 157 women (51%). Ages ranged from 19 to 41 years *(M* = 31.19, *SD* = 5.56, *Mdn* = 31). There were 17 dating individuals (5.5%), 125 individuals in a committed relationship (40.7%), and 165 married individuals (53.7%). Three participants (1%) indicated completing only up to primary school, 116 (37.8%) up to high school, 178 (56%) attained up to a bachelor’s or master’s degree, five individuals (1.6%) attained a Ph.D. degree, and five participants (1.6%) indicated ‘other’ level of education. All participants passed the attention check, implemented to control for the quality of engagement and enable the exclusion of respondents who did not provide meaningful responses; thus, all participants’ data were included in analyses.

### Measure

Participants completed the shortened version of the Triangular Love Scale (TLS-15), consisting of 15 items (i.e., 5 items comprising each of the Intimacy, Passion, and Commitment subscales). Responses ranged from 1–*not at all* to 5–*extremely*.

### Procedure

Participants were recruited with the help of an external company and were compensated for their participation. The study was conducted online using the Qualtrics web platform.

### Statistical Analyses

We followed a similar path of analysis as in Study 1. We first tested the basic psychometric characteristics of all three subscales and the total TLS-15 score, and proceeded with the CFA and IRT. All analyses were performed in R (4.1.0), with packages listed in the SM.

### Results

Table S10 in the SM presents means, standard deviations, skewness and kurtosis values, and reliability scores of the three love subscales and the total TLS-15 score. Reliability scores include Cronbach’s alpha, omega total, omega hierarchical, and EAP trait scores estimated from the GPCM models. In short, we found evidence of good reliability for all three love subscales (Cronbach’s alpha for Intimacy = 0.89, Passion = 0.89, Commitment = 0.92, and the entire TLS-15 = 0.95). Table S11 in the SM shows all item means, standard deviations, skewness values, and kurtosis values. In summary, skewness and kurtosis values for all items were within the expected range of the normal distribution and had more normal-shaped distributions than the corresponding items from the study by Sorokowski et al. (2021) based on the TLS-45. Item-subscale correlations were significant and varied from 0.79 to 0.87 for Intimacy (*M* = 0.84, *SD* = 0.04, *Mdn* = 0.85), from 0.78 to 0.88 for Passion (*M* = 0.84, *SD* = 0.03, *Mdn* = 0.84), and from 0.84 to 0.91 for Commitment (*M* = 0.87, *SD* = 0.03, *Mdn* = 0.87). Table S12 shows item-subscale correlations, while Table S13 in the SM shows inter-item correlations within the TLS-15 (which varied from 0.38 to 0.79, *M* = 0.58, *SD* = 0.08, *Mdn* = 0.58).

CFA yielded similar results for all three subscales separately and the three-factor model of TLS-15. Robust CFI and TLI values were all above 0.95, while robust RMSEA was below 0.08, and robust SRMR was below 0.06 (see Table S14 in the SM for detailed results of the CFA), which indicated good model fit. Item loadings were high and fell between 0.71 and 0.89 (*M* = 0.81, *SD* = 0.06, *Mdn* = 0.81). Details of item loadings are presented in Table S15 in the SM. The correlations between the three factors were: *r* = 0.89 between intimacy and passion, *r* = 0.87 between intimacy and commitment, and *r* = 0.82 between passion and commitment.

Next, we proceeded with the IRT analyses. First, we analyzed unidimensionality. The parallel analyses suggested one factor in the case of intimacy, passion, and commitment (analyzed separately) and a three-factor structure in the TLS-15 (analyzed jointly). Velicer’s (1976) minimum average partial (MAP) criterion indicated one factor within intimacy, passion, and commitment and two factors within the TLS. The ratio of the first to second eigenvalues (greater than 4:1) provided evidence for a one-factor structure of all three subscales analyzed separately. Adjusted Q3 statistics (aQ3) indicated that no items of passion, but two items of intimacy (one pair) and two items of commitment (one pair) might be considered as locally dependent. We then investigated whether a five-point scale is ordered. The evaluation of thresholds revealed that all items were ordered, except for the item 3 in intimacy subscale, which strongly suggests that the narrowed response scale should be retained (Silvia & Rodriguez, 2020). Figures S52–S66 in the SM show item characteristic curves.

All items had similar mean-square infit statistics, but they differed regarding the outfit statistics. In the case of the intimacy subscale, item outfit statistics ranged from 0.79 to 1.02; for passion from 0.91 to 1.06; and for commitment from 0.78 to 0.99. Item parameters and infit and outfit characteristics are presented in Tables S16 and S17, respectively. The evaluation of the RMSD statistics provided evidence for the proper fit of the items. All items’ RMSD statistics were below 0.05, ranging from 0.02 to 0.05 for intimacy (*M* = 0.03, *SD* = 0.01, *Mdn* = 0.02), from 0.02 to 0.04 for passion (*M* = 0.03, *SD* = 0.01, *Mdn* = 0.03), and from 0.01 to 0.05 for commitment (*M* = 0.03, *SD* = 0.01, *Mdn* = 0.03; see Table S18 in the SM for details).

The analyses of item difficulty and discrimination parameters are presented in Figures S67–S68 in the SM, respectively. The endorsement of all items was high. The *β* parameters of all items fell below 0 (ranging from –5.89 to –0.84, *M* = –1.57, *SD* = 1.17, *Mdn* = –1.29), meaning that the below-average level of the latent trait was sufficient to endorse the items. Passion was the most difficult subscale, while Intimacy and Commitment were less so. Notably, all subscales were rather easily and highly endorsed. The analysis of item discrimination revealed that most items had high discrimination parameters, ranging from 1.90 to 6.18 (*M* = 3.04, *SD* = 1.02, *Mdn* = 2.95).

Test-information functions revealed that the subscales differed regarding how much information they provided and at which trait levels they were the most reliable (see Figure S69 in the SM). The commitment and intimacy subscales provided much more information than the passion subscale, but they did so at a lower trait level than that of passion. Figure S69 reflects what can be drawn from discrimination and difficulty analyses: intimacy and commitment were easier but more discriminating than passion, while passion was more difficult and also less discriminative than intimacy and commitment. All items seemed to carry the most information at the lower end of the trait continuum. At the same time, all items were less discriminative for people with average and above-average levels of the trait. Total information function graphs show that the reliability of the subscales was above the minimal acceptable value of 0.70 (Taber, 2018), between –3.0 and 1.5 standard scores in the case of intimacy and commitment, and between –3.0 and 2.0 in the case of passion (see Figures S70–S72 in the SM).

### Discussion

Results of Study 2 supported the claim of good psychometric properties of the TLS-15 (see Table 2). All three love subscales (i.e., intimacy, passion, and commitment) were successfully re-created with their corresponding five items. Also, the three-factor structure of the TLS-15 was established. In the next step, we further tested the psychometric characteristics of the TLS-15, that is, test–retest reliability and convergent validity.

**Table 2:** Items from the 15-item Triangular Love Scale (TLS-15) with a 5-point response range (from 1–Not at all to 5–Extremely)

TLS-15
*Intimacy*
1. I have a warm relationship with my partner
2. I receive considerable emotional support from my partner
3. I value my partner greatly in my life
4. I have a comfortable relationship with my partner
5. I feel that my partner really understands me
*Passion*
6. My relationship with my partner is very romantic
7. I find my partner to be very personally attractive
8. I cannot imagine another person making me as happy as my partner does
9. There is something almost “magical” about my relationship with my partner
10. My relationship with my partner is passionate
*Commitment*
11. I have confidence in the stability of my relationship with my partner
12. I view my commitment to my partner as a solid one
13. I am certain of my love for my partner
14. I view my relationship with my partner as permanent
15. I feel a sense of responsibility toward my partner

## Study 3

Study 3 aimed to assess the test–retest reliability and convergent validity of the TLS-15 as compared to the TLS-45. For this goal, a within-subject repeated-measure design (with a two-week delay) was implemented. Half the participants completed the TLS-15 in the first wave and the same TLS-15 in the second wave, while the other half completed the TLS-45 in both waves. Importantly, to test for convergent validity, we used the same scales as Sternberg (1997) in his validation of the original TLS-45.

### Method

The Principal Investigator’s Institutional Review Board (IRB) at the Institute of Psychology, University of Wrocław approved the study’s protocol. All participants provided informed consent to participate in the survey.

### Power Analysis

Power analysis was conducted to determine the number of participants for Study 3. Using R (version 4.0.1), simulations of the obtained power to detect the assumed test–retest reliability were performed. In these analyses, a power of 80% was assumed for two measurements, a minimum reliability (cut-off point) of 0.7, an alpha of 0.05, and a correlation between measurements of 0.8. A bootstrapping analysis with 5,000 iterations indicated that the minimum number of participants in the second wave should be 122 (for each of the TLS versions). Based on the recruitment company’s experience, it was predicted that of those who would participate in the first wave, approximately 61% could be expected to also participate in the second wave. Hence, a minimum of 200 individuals had to be invited to participate in the first wave for both the TLS-15 or TLS-45, for a total minimum number of 400 individuals in the first wave, of whom 61% (*n* = 244, 122 per condition) were predicted to participate in the second wave.

### Participants

We recruited 413 Polish participants, among whom 197 self-reported as male (48%) and 215 as female (52%); one person did not indicate their sex. Age ranged between 18 and 65 years (*M* = 37.68, *SD* = 12.05, *Mdn* = 36). Of the total, 125 individuals were dating (30%), 38 were in a committed relationship (9%), and 250 were married (61%). The mean length of the relationship was 142.25 months (*SD* = 121.12). Regarding the level of education, 15 participants (4%) indicated completing only primary school, 203 (49%) up to high school, and 95 (56%) attained a university degree. All participants passed the attention check and were thus included in further analyses. In the first wave, part of the sample (*N* = 205) completed the TLS-15, while the other (*N* = 208) completed the TLS-45. Of the 413 participants from the first wave, 256 participated in the second wave of the study, of whom 134 re-completed the adapted TLS-15 scale and 122 re-completed the original TLS-45 scale.

### Procedure

Participants were recruited with the help of an external company and were compensated for their participation. The study was conducted online. In the first wave, each participant completed Rubin’s (1970) Love Scale and the Kansas Marital Satisfaction Scale (KMSS) (Schumm et al., 1983, 1986). In addition, half the participants completed the shortened Triangular Love Scale (TLS-15), while the other half completed the original Triangular Love Scale (TLS-45).

### Measures

Rubin’s Love Scale consists of 13 questions with a 9-point response scale (ranging from 1–*Definitely no* to 9–*Definitely yes*). An exemplary item from the scale reads, “I would do almost anything for my partner.” The scale has been validated in several studies (e.g., Amelang & Pielke, 1992; Dermer & Pyszczynski, 1978). The KMSS consists of three questions on a 7-point response scale (ranging from 1–*I am extremely dissatisfied* to 7–*I am extremely satisfied*). An exemplary item from the scale reads, "How satisfied are you with your relationship?” The KMSS has been used in many cross-cultural studies, as it exhibits good psychometric properties (e.g., Sorokowski et al., 2019).

### Statistical Analyses

Again, we followed a similar path of analysis as in Studies 1 and 2. We first tested the basic psychometric characteristics of all three subscales of the TLS-15 and TLS-45. In the next step, using the proportion test and Student’s *t*-test, we investigated whether there were differences (in terms of sex and relationship duration) between those who filled out the TLS-15 and TLS-45. The intraclass correlation coefficients (*ICC*) were then determined for the TLS-15 and TLS-45. Convergent validity was tested via Pearson’s correlations with the total score of Rubin’s Love Scale and the KMSS. All analyses were performed in R (4.1.0). All packages used are listed in the SM.

### Results

There were no differences in sex and relationship length between participants who completed the TLS-15 and TLS-45 (*x*^*2*^_*(1)*_ = 0.11, *p* = 0.92; *t*_*(411)*_ = 0.19, *p* = 0.85, respectively). Table S19 in the SM presents means, standard deviations, skewness values, kurtosis values, and reliability scores of all the scales from both waves. In short, both the TLS-15 and TLS-45 subscales were highly reliable. Interestingly, the TLS-45 had higher skewness and kurtosis values than the TLS-15. Convergent validity of the TLS-15 and TLS-45 was confirmed, with both scales showing significant correlations with Rubin’s Love Scale and the KMSS (see Table S20 in the SM). As indicated by 95% confidence intervals, these relationships did not differ between the TLS-15 and TLS-45, except for the correlation between Rubin’s Love Scale and passion, which was slightly stronger in the case of the TLS-45. Furthermore, there were no differences in correlations between the TLS-15 and the TLS-45 from the original Sternberg’s (1997) study (see Table S20 in the SM), except for commitment of the TLS-15, which showed a stronger correlation with the KMSS compared to the TLS-45. Furthermore, ICC revealed that both the TLS-15 and TLS-45 were reliably stable (see Table S21 in the SM). However, the TLS-45 was slightly more reliable in the case of passion and commitment (a gap of 0.01 and 0.07 in 95% CI, respectively).

### Discussion

The results of Study 3, a repeated-measures study validating both the TLS-15 and TLS-45, confirmed convergent validity of the TLS-15 with that of the original version and other indices of love. The correlations between the TLS-15 and both Rubin’s (1970) Love Scale and the Kansas scale (Schumm et al., 1983, 1986) did not differ markedly compared with the correlations between the TLS-45 from Study 3 and from the original Sternberg (1997) study and the same validity scales. The only two exceptions were: passion was slightly more strongly related to the Rubin’s Love Scale for the TLS-45 than for the TLS-15 in Study 3, and commitment was slightly more strongly related to the KMSS for the TLS-15 than for the TLS-45 from the Sternberg (1997) study. In addition, the TLS-15 had good test–retest reliability (Fleiss, 1986; Oremus et al., 2012), as indicated by the ICC. Nevertheless, although the ICC of the TLS-15 and TLS-45 did not differ for intimacy and passion (as indicated by the confidence intervals), commitment of the TLS-45 had a higher ICC and, thus, was more stable than commitment of the TLS-15. After confirming the good psychometric properties of the TLS-15 in Studies 2 and 3, we proceeded with a large, cross-cultural investigation in Study 4 that was part of a larger research project focused on love, mate attraction, and physical attractiveness (see e.g., Kowal et al., 2022).

## Study 4

In Study 4, we aimed to extend the results of the previous three studies on a larger cross-cultural sample and to test whether the short version of the Triangular Love Scale (TLS-15) holds good psychometric properties. A further goal was to test the cross-cultural validity of different linguistic versions of the TLS-15.

### Method

The study was conducted in the framework of a cross-cultural investigation with 404 contributing scholars from 105 participating countries. The Principal Investigator’s Institutional Review Board (IRB) at the Institute of Psychology, University of Wrocław, approved the study’s protocol. All collaborators who collected data followed the ethical guidelines of their IRBs, acting either on the ethical approval of the Principal Investigator’s IRB or ethical approval received from their local IRB. All participants provided informed consent to participate in the survey.

### Power Analysis

The power required in cross-cultural studies and in invariance equivalence analyses is difficult to estimate. It often causes heated debates (Matsumoto et al., 2010; van de Vijver & Leung, 2021). Some researchers suggest a minimum of 200 participants per group (Kline, 2005), while others advise a minimum of 93 participants (van de Vijver et al., 2019). Based on indications in the literature and our own experience in cross-cultural data analysis (Ikizer et al., 2022; Kowal et al., 2020a, 2020b, 2021, 2022), we decided to test the equivalence of invariance on a minimum number of 100 participants per linguistic group.

### Participants

A total of 118,320 participants from 175 countries responded to the recruitment call and completed the survey in one of the 43 available languages. Of those, data from 60,311 adult individuals across 156 countries were processed for further analysis, including 41,447 (69%) female participants, 18,169 (30%) male participants, 449 (0.7%) non-binary participants, and 246 (0.4%) who preferred not to indicate their sex. Participants were aged between 18 and 90 years (*M* = 32.41, *SD* = 12.89, *Mdn* = 28). Inclusion in the analyses was based on the following criteria: participants reported that they were romantically involved, they passed the attention check providing meaningful answers, and there were at least 100 responses per language version of the survey. Six languages (Bengali, Swahili, Urdu, Chinese-simplified, Mongolian, and Thai) did not pass this latter inclusion criterion. The following 37 languages were included in our study: Arabic, Bosnian, Brazilian Portuguese, Bulgarian, Chinese Traditional, Croatian, Czech, Dutch, English, Estonian, Farsi, Finnish, French, Georgian, German, Greek, Hebrew, Hungarian, Italian, Japanese, Korean, Lithuanian, Macedonian, Malaysian, Norwegian, Polish, European Portuguese, Romanian, Russian, Serbian, Slovak, Slovenian, Spanish, Spanish (Latin), Swedish, Turkish, and Ukrainian (see Table S22 in the SM). The respondents were either dating someone (13,180, 22%), in a committed relationship (25,755, 43%), or married (21,376, 35%). In all, 145 participants (0.2%) indicated having no formal education, 263 (0.4%) completed only up to primary school, 2743 (4.6%) completed up to secondary school, 10,968 individuals (18.2%) completed up to high school, 21,550 (35.7%) attained up to a bachelor degree, 11,885 (19.7%) attained up to a master degree, and 3668 (6.1%) attained a doctoral degree; 9089 participants (15.1%) did not respond to the question about their education level. A detailed description of participants per country is given in Table S23 in the SM.

### Procedure

We forward- and back-translated (Brislin, 1970, 1983) the survey into 43 languages (see SM for detailed instructions for all translating teams). Data collection spanned five months (April–August 2021). Data were collected online via the Qualtrics platform in all but four countries. One Russian collaborator collected data using the Toloka website (a crowd-sourcing platform popular in Russia). Individuals from Algeria and Morocco could not access the Qualtrics site, so data were collected in person using a paper-and-pencil method. Iranian participants also had difficulties accessing the Qualtrics site, so data were collected using Google Forms. Collaborators invited participants from as diverse sample pools as possible (e.g., men and women, older and younger, from rural and urban areas, from the community and university samples). Participants were also asked to share the link to the survey on their social media. Approximately 6% of the data were collected with the help of outsourcing companies.

### Statistical Analyses

In the first step, we followed the statistical approach from the previous studies and calculated the basic psychometric properties of the TLS-15 (i.e., reliabilities and correlations). We then proceeded with multigroup confirmatory factor analyses to establish the equivalence of the TLS-15 across languages. After testing for configural invariance, we constricted the factor loadings (metric invariance) and intercepts to be equal (scalar invariance). Model comparisons were assessed following the recommended criteria, including a change of CFI (ΔCFI) and TLI (ΔTFI) no greater than 0.01, a change of RMSEA (ΔRMSEA) no greater than 0.015, and a change of SRMR (ΔSRMR) no greater than 0.01, indicating that the two compared models did not differ in terms of model fit (Chen, 2007; Cheung & Rensvold, 2002). In the last step, we applied IRT analysis with the GPCM model. All analyses were performed in R (4.1.0). All packages used are listed in the SM.

### Results

Table S24 presents means, standard deviations, skewness, kurtosis, and reliability values (Cronbach’s alpha, omega total, omega hierarchical, and expected a posteriori EAP trait scores, which were estimated from the GPCM models) of the three love subscales separately and jointly, in a three-factor solution. In summary, all items were within the expected range of normality distribution. Furthermore, we found evidence for the good reliability of the three love subscales. Table S25 in the SM shows means, standard deviations, Cronbach’s alpha, and McDonald’s omega for each of the languages. Item-subscale and inter-item correlations showed that all items correlated with each other (more so within the subscale; see Tables S26 and S27 in the SM, respectively). Item-subscale correlations were significant and varied from 0.81 to 0.87 for Intimacy, from 0.76 to 0.86 for Passion, and from 0.72 to 0.87 for Commitment.

In the next step, we conducted multigroup confirmatory factor analyses. The analyses indicated that the overall model with a three-factor structure (TLS-15) fit the data well regarding configural, metric, and scalar invariance (see Table S28 in the SM). However, although the ΔCFI and ΔTLI were below the cut-off of 0.01 after constricting loadings and intercepts (ΔCFI = 0.007, ΔTLI = 0.006 and ΔCFI = 0.008, ΔTLI = 0.007, respectively), as well as ΔRMSEA (ΔRMSEA = 0.013 and ΔRMSEA = 0.009, respectively), ΔSRMR was above the cut-off (ΔSRMR = 0.017 and ΔSRMR = 0.008, respectively). Hence, we decided to constrain loadings and intercepts of two items from each scale (i.e., Intimacy items 1 and 3, Passion items 7 and 8, Commitment items 13 and 15) to test for a partial metric and partial scalar invariance (Byrne et al., 1989; Steenkamp & Baumgartner, 1998). The analysis of partial metric and partial scalar invariance provided evidence that such models can be considered invariant (ΔCFI = 0.003, ΔTLI = 0.003, ΔRMSEA = 0.007, ΔSRMR = 0.009 between configural and partial metric, and ΔCFI = 0.003, ΔTLI = 0.003, ΔRMSEA = 0.005, ΔSRMR = 0.004 between partial metric and partial scalar). We repeated the above steps for each of the love components (i.e., Intimacy, Passion, and Commitment; see Table S28 in the SM). Similarly, as in the TLS-15 case, partial scalar invariance was reached in all three cases. The common variance explained by the three-factor structure CFA model was 62%. Based on the analysis of the BIC and AIC, we inferred that the hierarchical three-factor structure was superior to the one-factor model that captured all 15 items (ΔBIC = 43,695, ΔAIC = 43,722).

**Fig. 1:**
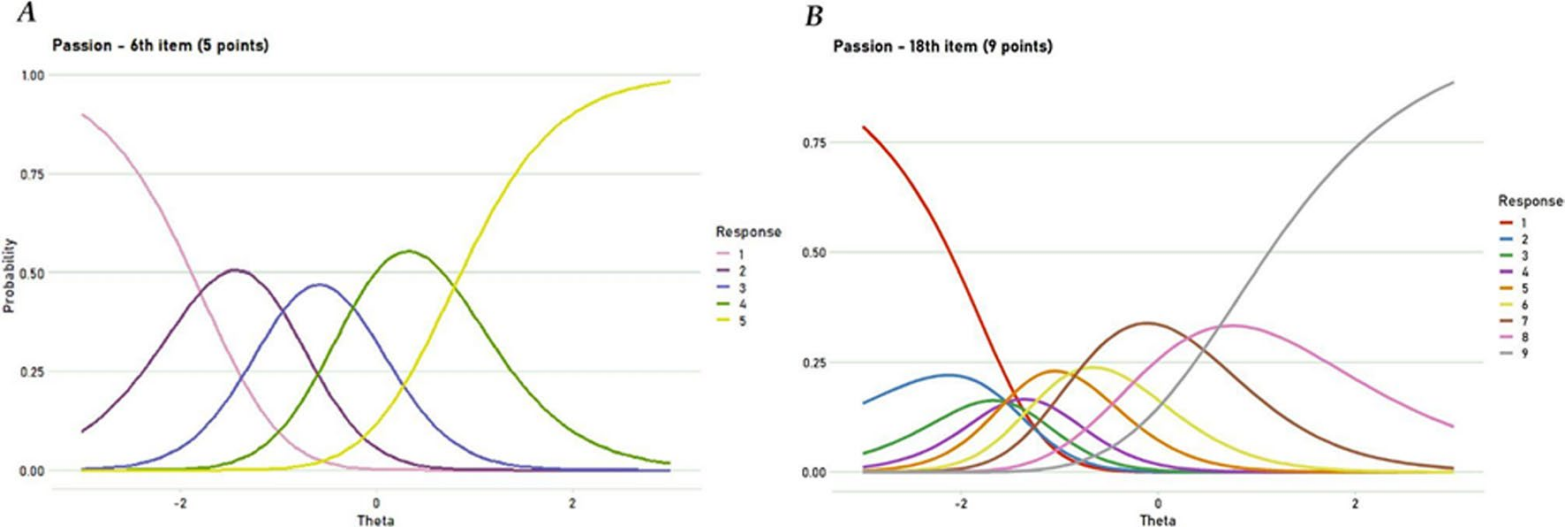
A graphical representation of item thresholds of the first item from the passion subscale from the TLS-15 (with a 5-point item response range, **a**) and the corresponding item from the TLS-45 (with a 9-point item response range, **b**). *Note.* This comparison is made on two different samples, so any conclusions warrant caution

Next, we proceeded with the IRT analyses. First, we conducted the analysis of unidimensionality. Although the parallel analyses suggested three factors per subscale, the first factor was clearly dominant. The minimum average partial (MAP) criterion and the ratio of the first to second eigenvalues (greater than 4:1) also provided evidence for a one-factor structure of all three subscales tested separately. Adjusted Q3 statistics revealed that within the subscales, all items were locally independent. The visual inspection of the item characteristic curves revealed that all items might be considered as ordered. Figure 1 illustrates an exemplary item from Study 1 and the corresponding item from Study 4 with an altered response range (the visualization of all items can be found in SM, Figures S73–S87). A summary of the parameters from the GPCM is presented in Table S29 (SM).

All items showed no infit and outfit (i.e., these statistics were close to 1.00, see Table S30 in the SM). The evaluation of the RMSD misfit statistics confirmed the items’ goodness of fit (Table S31 in the SM). All items’ RMSD were below 0.05, ranging from 0.02 to 0.03 (*M* = 0.02, *SD* = 0.004, *Mdn* = 0.02). The analysis of items’ difficulty and discrimination is presented in Figures S88–S89, accordingly. Similarly to Studies 1 and 2, the endorsement of all items was high, as the *β* parameter of all items fell below 0. The most difficult subscale was Passion, with the exception of the seventh item, while Intimacy and Commitment were less difficult. The analysis of item discrimination revealed that most of the discrimination values were fairly high, ranging from 1.17 to 3.12 (*M* = 2.09, *SD* = 0.54, *Mdn* = 2.12). Figure S90 in the SM reflects what can be drawn from discrimination and difficulty analyses: intimacy and commitment were more endorsed and more discriminative than passion, while passion was less endorsed and less discriminative than intimacy and commitment.

Test information functions revealed that subscales differed regarding how much information they provided and at which trait levels they were most reliable. The commitment and intimacy subscales provided more information than the passion subscale, but did so at a slightly lower trait level than passion. Nevertheless, all items seemed to have provided the most information at the below-average trait levels. Total information function graphs show that the reliability of the subscales was above the minimal acceptable value of 0.70 (Taber, 2018), between –3 and 1.5 standard scores in the case of intimacy and passion and between –3 and 1 in the case of Commitment (see Figures S91–S93 in the SM).

### Discussion

Study 4 supported the results of previous studies. Good psychometric properties of the TLS-15 were confirmed in a large cross-cultural sample (*N* = 60,311). Furthermore, the multigroup CFA revealed that the 37 linguistic versions of the TLS-15 may be considered invariant, allowing for score comparisons across cultures and countries.

## General Discussion

Our aim was to investigate whether the 45-item Triangular Love Scale (Sternberg, 1997), commonly used to assess levels of romantic love in people who are in a romantic relationship, could be shortened while retaining its original three-factor structure as well as validly and reliably measuring experiences of romantic love. We not only achieved this methodological goal, but also provided evidence that the Triangular Theory of Love can be validated across 37 languages with distinct cultural backgrounds, yielding comparable results.

In Study 1, based on a re-analysis of data from a large-scale study of 7332 individuals using the original version of the TLS-45 (Sorokowski et al., 2021), we selected 15 items demonstrating the best psychometric properties based on kurtosis, skewness, item-scale correlations, CFA loadings, misfit statistics, and parameters of difficulty and discrimination. This included five items from each of the love components: intimacy, passion, and commitment. Results revealed that the TLS-15 successfully re-created the structure of the TLS-45. We found evidence that the original scale’s 9-point structure might be inappropriate, as item thresholds were disordered. We thus revised the TLS-15 to include 5-point scales. The main shortcoming of Study 1 was that, when checking the psychometric properties of the TLS-15, we utilized data drawn from participants who answered all 45 items of the TLS-45. It imposed a risk that the remaining 30 items (and their configuration) could affect how participants responded.

To address these limitations, we conducted Study 2, which confirmed that the TLS-15 can stand alone and provide comparable information to the TLS-45. The three-factor structure was well represented within the selected 15 items, with evidence that the 5-point response format was indeed more appropriate, as item thresholds were more ordered. The aim of Study 3 was to measure the convergent validity and test–retest reliability of the TLS-15 compared with the TLS-45. We observed high correlations between the TLS-15 and both Rubin’s Love Scale and the KMSS, comparable to those of the TLS-45, which confirmed the convergent validity of the TLS-15. Furthermore, the results confirmed the test–retest reliability, that is, stability of the TLS-15 scores across time.

The final, large-scale Study 4, conducted on 60,311 participants from 156 countries, confirmed the results of the previous three studies, further underscoring the cross-cultural validity and applicability of the TLS-15. The 15 items effectively recreated the three-factor structure and the one-factor structure of each of the three love components. This means that it is possible to use the TLS-15 to measure love based on three love components (i.e., intimacy, passion, and commitment), as well as to use the three love components separately (e.g., only to apply the first five items of the scale to measure intimacy, the following five items to measure passion, and the last five items to measure commitment). Study 4 also provided evidence for the partial scalar invariance across 37 linguistic versions of the TLS-15, indicating its applicability in cross-cultural research. Finally, the shortened, 5-point response format was found to be superior to the original 9-point range, as it showed better monotonicity within the corresponding items.

Notably, we validated a short Triangular Love Scale (TLS-15) derived from the same Triangular Love Theory as the original TLS-45. We did not change the items’ wording, so the concept of three love components, namely intimacy, passion, and commitment (Sternberg, 1988), remains intact. This is essential because the triangular theory of love is one of the most popular conceptions of love (Hatfield et al., 2012) and, by some, even considered the gold standard (Campbell & Kaufman, 2017). Indeed, the three factors of the TLS-45 yield highly reliable scores across diverse samples (e.g., Graham & Christiansen, 2009). In Graham’s (2011) meta-analysis, Intimacy, Passion, and Commitment from the TLS-45 had the highest loadings on the love factor (compared to LAS, PLS, and Rubin’s Love Scale).

Furthermore, the results of Study 4 provided further evidence for the three-factor structure of the TLS-15. When we compared the two CFA models, we found that the one with a three-factor structure had a superior fit to the single-factor structure. This aligns with previous research that the three-factor model is adequate for the various modifications of the TLS (e.g., Gouveia et al., 2009; Overbeek et al., 2007). On the other hand, we found high correlations between Intimacy, Passion, and Commitment. Thus, our results also support previous concerns over the distinctiveness of the three love factors (e.g., Graham, 2011; Hendrick & Hendrick, 2003; Merino & Privado, 2020). It is reasonable to assume that love components are inextricably linked. However, it would be illuminating to establish what exactly drives these interrelationships and to further elaborate on the discriminative validity of these subscales. It could translate into disentangling these relationships and creating more unique items within the TLS-15.

Love, as a multifaceted construct, has long captivated the interest of researchers, philosophers, and artists alike. It seems understandable—there is mounting evidence that love affects almost all aspects of our lives, from mating (Buss, 1989) to psychological well-being (Kansky, 2018; Oravecz et al., 2020), happiness (Tamir et al., 2017), and health (Fletcher et al., 2015). Love’s influence also spans the social environment. Many studies have found love’s dyadic or family-level impact on, for instance, partners’ mental and physical health (Gallacher & Gallacher, 2011), healthy (and unhealthy) habits (Jackson et al., 2015; Keller et al., 2020), and children’s development and achievements (Amato & Keith, 1991; Auersperg et al., 2019).

Our research also supports the universality of romantic love experiences viewed from the lens of the Triangular Love Theory (Sternberg, 1988, 2021). As evidenced by Study 4, 60,311 individuals from 156 countries experienced comparable levels of intimacy, passion, and commitment toward their partners. This observation not only reinforces the robustness of the theory’s conceptual framework, but also emphasizes the human capacity to feel romantic love. This capacity transcends cultural, linguistic, societal, and geographical boundaries.

We believe that love is essential, and future studies should be devoted to investigating the love phenomenon. We hope that the proposed TLS-15 will help us achieve greater consensus on how we measure love and thus contribute to advances in its study. However, we wish to emphasize that the TLS-15 carries the most information at the lower levels of love, so it can more reliably differentiate between individuals experiencing average and below-average levels of love than between individuals of above-average love experiences. This may be crucial from a clinical or psychological perspective, as the TLS-15 might be especially useful for couple counselors and therapists when working with couples with relationship issues, often those who experience lower levels of love (Kurdek, 2002).

Although our article has been primarily written from the perspective of the researcher, there are two other perspectives—those of counselors or therapists and those of clients—that are important to understanding why a shorter version of the Triangular Love Scale will be useful in practice, as well as in theory and research. First, for those counselors who use the scale to assess love or compatibility, it is advantageous to have a shorter scale whose administration can better fit into the usual 50-min therapy or counseling “hour.” With the need for preparation, set-up, and possibly scoring, the shorter version is much more practical for counseling or therapeutic purposes. Second, clients taking the assessment will be less burdened by a 15-item assessment than by a 45-item one. The shorter assessment reduces the chances of test fatigue and may also provide better data for end-of-scale questions that otherwise might have been given shorter shrift.

Perhaps most crucial is that we have provided a psychometrically validated, abbreviated assessment that can ensure compatibility across different uses of the Sternberg triangular love scale to the maximum extent possible. In the past, investigators have used different purported versions of the same test; these provide no guarantee of measuring the same thing as each other, nor what is measured by the longer version.

Although our article provides evidence for the usefulness of the TLS-15, it is not without limitations. First, in our studies, we focused on the triangular love theory (Sternberg, 1988), and thus, we only included the triangular love scale. The evidence of the relationships between various love scales is equivocal (e.g., Graham, 2011; Masuda, 2003), and thus, future research could shed more light on the comparison of the TLS with other love scales (such as the passionate love scale or the love attitudes scale). Second, as the focus of this study was on the factor and convergent validity of the TLS-15, future research may explore other types of validity (e.g., discriminative, predictive, or diagnostic validity). Third, as mentioned above, the TLS-15 does not differentiate high levels of love (or any of the love components) as well as might be hoped. This is to be expected, as any study of love among individuals in relationships, by definition, is limited to measuring love among those who remain in relationships and have not split up. Hence, such individuals usually tend to view their relationship as at least somewhat satisfactory. Nevertheless, further studies could attempt to modify the questions’ content so that the items could better distinguish individuals with average levels of love components. Fourth, the TLS-15 was only validated on individuals in a romantic relationship (i.e., dating, in a committed relationship, or married), while the original TLS-45 also captures other types of love (e.g., motherly love). More research is needed to establish whether the TLS-15 can also be used in different types of relationships (e.g., polyamorous relationships, or relationships other than romantic contexts), thereby adjusting for the recipient of the love.

### Conclusions

In summary, the present article validated a short version of the Triangular Love Scale (TLS-15) that consists of 15 items, five items per scale (intimacy, passion, commitment), with a five-point response range (from 1–*not at all* to 5–*extremely*). The proposed scale has 37 cross-cultural invariant versions (including Arabic, Bosnian, Brazilian Portuguese, Bulgarian, Chinese Traditional, Croatian, Czech, Dutch, English, Estonian, Farsi, Finnish, French, Georgian, German, Greek, Hebrew, Hungarian, Italian, Japanese, Korean, Lithuanian, Macedonian, Malaysian, Norwegian, Polish, European Portuguese, Romanian, Russian, Serbian, Slovak, Slovenian, Spanish, Latin Spanish, Swedish, Turkish, and Ukrainian), which can be used in future studies (see SM, accessed under the link https://osf.io/sazfc/). Furthermore, our study is the first to validate a theory of love with such distinct cross-cultural groups, providing evidence that it is possible to yield comparable results across so many languages and cultures.

## Data Availability

All data have been made publicly available at the osf and can be accessed at https://osf.io/sazfc/. Analysis code for this study is available by emailing the corresponding author.
