# Correction to: Validation of the Short Version (TLS-15) of the Triangular Love Scale (TLS-45) Across 37 Languages

**DOI:** 10.1007/s10508-023-02751-y

**Published:** 2023-12-01

**Authors:** Marta Kowal, Piotr Sorokowski, Bojana M. Dinić, Katarzyna Pisanski, Biljana Gjoneska, David A. Frederick, Gerit Pfuhl, Taciano L. Milfont, Adam Bode, Leonardo Aguilar, Felipe E. García, S. Craig Roberts, Beatriz Abad-Villaverde, Tina Kavčič, Kirill G. Miroshnik, Izuchukwu L. G. Ndukaihe, Katarína Šafárová, Jaroslava V. Valentova, Toivo Aavik, Angélique M. Blackburn, Hakan Çetinkaya, Izzet Duyar, Farida Guemaz, Tatsunori Ishii, Pavol Kačmár, Jean C. Natividade, Ravit Nussinson, Mohd Sofian B. Omar-Fauzee, Ma. Criselda T. Pacquing, Koen Ponnet, Austin H. Wang, Gyesook Yoo, Rizwana Amin, Ekaterine Pirtskhalava, Reza Afhami, Alexios Arvanitis, Derya Atamturk Duyar, Théo Besson, Mahmoud Boussena, Seda Can, Ali R. Can, João Carneiro, Rita Castro, Dimitri Chubinidze, Ksenija Čunichina, Yahya Don, Seda Dural, Edgardo Etchezahar, Feten Fekih-Romdhane, Tomasz Frackowiak, Nasim Ghahraman Moharrampour, Talía Gómez Yepes, Simone Grassini, Marija Jovic, Kevin S. Kertechian, Farah Khan, Aleksander Kobylarek, Valerija Križanić, Samuel Lins, Tetyana Mandzyk, Efisio Manunta, Tamara Martinac Dorčić, Kavitha N. Muthu, Arooj Najmussaqib, Tobias Otterbring, Ju Hee Park, Irena Pavela Banai, Mariia Perun, Marc Eric S. Reyes, Jan P. Röer, Ayşegül Şahin, Fatima Zahra Sahli, Dušana Šakan, Sangeeta Singh, Sanja Smojver-Azic, Sinem Söylemez, Ognen Spasovski, Anna Studzinska, Ezgi Toplu-Demirtas, Arkadiusz Urbanek, Tatiana Volkodav, Anna Wlodarczyk, Mohd Faiz Mohd Y. Yaakob, Mat Rahimi Yusof, Marcos Zumárraga-Espinosa, Maja Zupančič, Robert J. Sternberg

**Affiliations:** 1 IDN Being Human Lab, University of Wrocław, Dawida 1, 50-529 Wrocław, Poland; 2 Institute of Psychology, University of Wrocław, Wrocław, Poland; 3 Department of Psychology, Faculty of Philosophy, University of Novi Sad, Novi Sad, Serbia; 4 ENES Bioacoustics Research Lab, Centre de Recherche en Neurosciences de Lyon, University of Jean Monnet Saint Étienne, Saint Étienne, France; 5 Centre National de la Recherche Scientifique, Laboratoire Dynamique du Langage, University of Lyon, Lyon, France; 6 Macedonian Academy of Sciences and Arts, Skopje, North Macedonia; 7 Crean College of Health and Behavioral Sciences, Chapman University, Orange, CA USA; 8 Department of Psychology, Norwegian University of Science and Technology, Trondheim, Norway; 9 School of Psychology, University of Waikato, Tauranga, New Zealand; 10 School of Archaeology and Anthropology, ANU College of Arts and Social Sciences, The Australian National University, Canberra, Australia; 11 School of Psychology, Central University of Venezuela, Caracas, Venezuela; 12 Division of Psychology, University of Stirling, Stirling, UK; 13 Departamento de Psiquiatría y Salud Mental, Facultad de Medicina, Universidad de Concepción, Concepción, Chile; 14 Faculty of Humanities and Education, Universidad Nacional Pedro Henríquez Ureña, Santo Domingo, Dominican Republic; 15 Department of Psychology, Faculty of Arts, University of Ljubljana, Ljubljana, Slovenia; 16 Faculty of Psychology, Saint Petersburg State University, Saint Petersburg, Russia; 17 Department of Psychology, Alex Ekwueme Federal University, Ndufu-Alike, Nigeria; 18 Institute of Psychology, Czech Academy of Sciences, Brno, Czech Republic; 19 Department of Experimental Psychology, Institute of Psychology, University of Sao Paulo, Sao Paulo, Brazil; 20 Institute of Psychology, University of Tartu, Tartu, Estonia; 21 Department of Psychology and Communication, Texas A&M International University, Laredo, TX USA; 22 Department of Psychology, Yasar University, İzmir, Turkey; 23 Department of Anthropology, İstanbul University, İstanbul, Turkey; 24 Department of Psychology and Educational Sciences, University Mohamed Lamine Debaghine Setif2, Setif, Algeria; 25 Department of Psychology, Japan Women’s University, Tokyo, Japan; 26 Department of Psychology, Faculty of Arts, Pavol Jozef Šafárik University in Košice, Košice, Slovakia; 27 Department of Psychology, Pontifical Catholic University of Rio de Janeiro, Rio de Janeiro, Brazil; 28 Department of Education and Psychology, The Open University of Israel, Raanana, Israel; 29 Institute of Information Processing and Decision Making, University of Haifa, Haifa, Israel; 30 School of Education, Universiti Utara Malaysia, Sintok, Malaysia; 31 Department of Psychology, University of Santo Tomas, Manila, Philippines; 32 Faculty of Social Sciences, Ghent University, Ghent, Belgium; 33 Department of Political Science, University of Nevada, Las Vegas, Las Vegas, NV, USA; 34 Department of Child & Family Studies, Kyung Hee University, Seoul, Republic of Korea; 35 Department of Professional Psychology, Bahria University, Islamabad, Pakistan; 36 Department of Psychology, Ivane Javakhishvili Tbilisi State University, Tbilisi, Georgia; 37 Department of Art Studies, Tarbiat Modares University, Tehran, Iran; 38 Department of Psychology, University of Crete, Rethymno, Greece; 39 Laboratoire de Psychologie Sociale, Université Paris Cité, Paris, France; 40 Department of Psychology, İzmir University of Economics, İzmir, Turkey; 41 Department of Anthropology, Hatay Mustafa Kemal University, Hatay, Turkey; 42 Center for Psychology at the University of Porto, University of Porto, Porto, Portugal; 43 Institute of Psychiatry, Psychology & Neuroscience (IoPPN), Department of Psychological Medicine, King’s College London, London, UK; 44 Institute of Psychology, Vilnius University, Vilnius, Lithuania; 45 Department of Psychology, University of Buenos Aires, Buenos Aires, Argentina; 46 Department of Psychology, Centro Interdisciplinario de Psicología Matemática y Experimental, Buenos Aires, Argentina; 47 Department of Education, International University of Valencia, Valencia, Spain; 48 Department of Psychiatry Ibn Omrane, Razi Hospital, Manouba, Tunisia; 49 Faculty of Medicine of Tunis, Tunis El Manar University, Tunis, Tunisia; 50 School of Psychological Sciences, University of Melbourne, Melbourne, Australia; 51 Department of Psychosocial Science, University of Bergen, Bergen, Norway; 52 Cognitive and Behavioral Neuroscience Laboratory, University of Stavanger, Stavanger, Norway; 53 Department of Marketing Management and Public Relations, Faculty of Organizational Sciences, University of Belgrade, Belgrade, Serbia; 54 Department of Organization, Management, and Human Resources, ESSCA School of Management, Paris, France; 55 Institute of Education & Research, Women University Mardan, Mardan, Pakistan; 56 Department of Pedagogy, University of Wrocław, Wrocław, Poland; 57 Department of Psychology, Faculty of Humanities and Social Sciences, J.J. Strossmayer University of Osijek, Osijek, Croatia; 58 Department of Psychology, Ivan Franko National University of Lviv, Lviv, Ukraine; 59 Cognition, Langues, Langage, and Ergonomie, University of Toulouse, Toulouse, France; 60 Department of Psychology, Faculty of Humanities and Social Sciences, University of Rijeka, Rijeka, Croatia; 61 Department of Psychology and Counselling, Universiti Tunku Abdul Rahman, Kampar, Malaysia; 62 Department of Applied Psychology, National University of Modern Languages, Islamabad, Pakistan; 63 Department of Strategy, University of Agder, Kristiansand, Norway; 64 Department of Child and Family Studies, Yonsei University, Seoul, Korea; 65 Department of Psychology and Psychotherapy, Witten/Herdecke University, Witten, Germany; 66 Institute of Sport Professions, University of Ibn Tofail, Kenitra, Morocco; 67 Department of Psychology, Faculty of Law and Business Studies Dr Lazar Vrkatić, Union University, Novi Sad, Serbia; 68 Department of Strategy and Management, University of Agder, Kristiansand, Norway; 69 Department of Psychology, Manisa Celal Bayar University, Manisa, Turkey; 70 Department of Psychology, University of Ss. Cyril and Methodius, Trnava, Slovakia; 71 Department of Psychology, Ss. Cyril and Methodius University in Skopje, Skopje, Republic of North Macedonia; 72 Department of Humanities, Icam School of Engineering, Toulouse Campus, Toulouse Cedex, France; 73 Psychological Counseling and Guidance, Mef University, İstanbul, Turkey; 74 Department of Pedagogy and Psychology, Kuban State University, Krasnodar, Russia; 75 Escuela de Psicología, Universidad Católica del Norte, Antofagasta, Chile; 76 Psychology Career, Salesian Polytechnic University, Quito, Ecuador; 77 Department of Psychology, Cornell University, Ithaca, NY USA

**Correction to: Archives of Sexual Behavior** 10.1007/s10508-023-02702-7

The affiliation presented for coauthor Ekaterine Pirtskhalava was incorrect in the article as originally published.

The correct affiliation for Dr. Pirtskhalava is:

Department of Psychology,

Ivane Javakhishvili Tbilisi State University,

Tbilisi, Georgia

The original article has been corrected.

